# Dietary Considerations for Inflammatory Bowel Disease Are Useful for Treatment of Checkpoint Inhibitor-Induced Colitis

**DOI:** 10.3390/cancers15010084

**Published:** 2022-12-23

**Authors:** Aditi Saha, Isabella Dreyfuss, Humaira Sarfraz, Mark Friedman, Joseph Markowitz

**Affiliations:** 1 Department of Cutaneous Oncology, H. Lee Moffitt Cancer Center and Research Institute, Tampa, FL 33612, USA; 2 Department of Gastrointestinal Oncology, H. Lee Moffitt Cancer Center and Research Institute, Tampa, FL 33612, USA; 3 Department of Oncologic Sciences, University of South Florida School of Medicine, Tampa, FL 33612, USA

**Keywords:** checkpoint-induced colitis, inflammatory bowel disease, checkpoint blockade, cytotoxic T-lymphocyte-associated antigen 4 (CTLA-4), programmed cell death ligand 1 (PD-1), enteritis, diet

## Abstract

**Simple Summary:**

We will briefly review the pathophysiologic, dietary, and genetic factors associated with inflammatory bowel diseases, such as Crohn’s disease and ulcerative colitis. This review will elaborate on how the principles developed for the treatment of inflammatory bowel disease can be applied to patients experiencing inflammatory bowel toxicity secondary to checkpoint blockade. The medical oncology and gastroenterology perspectives are presented in this review.

**Abstract:**

Checkpoint molecules are cell surface receptors on immune cells that mitigate excessive immune responses, but they have increased expression levels in cancer to facilitate immune escape. Checkpoint blockade therapies (e.g., anti–PD-1, anti–CTLA-4, and anti–LAG-3 therapy, among others) have been developed for multiple cancers. Colitis associated with checkpoint blockade therapy has pathophysiological similarities to inflammatory bowel disease (IBD), such as Crohn’s disease and ulcerative colitis. Current therapeutic guidelines for checkpoint blockade-induced colitis include corticosteroids and, if the patient is refractory to steroids, immunomodulating antibodies, such as anti-TNF and anti-integrin agents. Interestingly, immunomodulatory molecules, such as TNFα, are upregulated in both IBD and checkpoint-mediated colitis. The inflammatory colitis toxicity symptoms from checkpoint blockade are similar to clinical symptoms experienced by patients with IBD. The pathophysiologic, dietary, and genetic factors associated with IBD will be reviewed. We will then explain how the principles developed for the treatment of IBD can be applied to patients experiencing inflammatory bowel toxicity secondary to checkpoint blockade.

## 1. Introduction

In the era of immunotherapy, antibodies directed against immune checkpoints are effective therapeutics in a variety of malignancies, including melanoma, lung carcinoma, colon cancer, and renal cell carcinoma [1,2,3]. For example, in melanoma, clinically actionable checkpoint inhibitors are directed against programmed death 1 (PD-1), cytotoxic T-lymphocyte-associated antigen 4 (CTLA-4), programmed death ligand 1 (PD-L1), and lymphocyte-activation protein 3 (LAG3) (Table 1) [4]. In normal biology, these checkpoint molecules control our immune response to prevent autoimmunity [5]. Monoclonal antibodies against these regulatory proteins (ICIs) promote our own immune system and increase the antitumor activity of cytotoxic T cells [3]. However, unrestrained activation of immune cells causes excessive inflammation in the body, and the resulting autoimmunity leads to immune-related adverse events (irAEs), including but not limited to colitis, hepatitis, pancreatitis, hypophysitis, dermatitis, nephritis, and pneumonitis. The incidence of checkpoint inhibitor induced colitis (CIC) ranges somewhat between 1% and 40% depending on the type of ICI used and the underlying malignancy [6,7].

Inflammatory bowel disease (IBD), encompassing Crohn’s disease (CD) and ulcerative colitis (UC), is a cytokine-derived chronic and relapsing small and large bowel inflammatory condition [8]. Similarities in pathophysiology, clinical presentation, immunological response, and pharmacologic treatment between IBD and CIC were previously published [9,10,11,12]. Besides different immunomodulatory medications, dietary modifications and the role of specific diets are well-studied for the prevention of flares in IBD. Here, we will review the pathological similarities between IBD and checkpoint inhibitor therapy-mediated colitis, with the goal of providing a perspective on the diets that may be used to prevent CIC or facilitate recovery.

**Table 1:** Relevant checkpoint molecules, cells that express these checkpoint molecules, and FDA-approved antibodies for the treatment of cutaneous malignancies.

Checkpoint Molecules	Cells Expressing Checkpoint Molecules	Checkpoint Antibodies
**PD-1** [13]	T cells	pembrolizumab nivolumab cemiplimab
	B cells	
	Monocytes	
	TAMs	
	NK	
	DC	
	MDSCs	
	Tumor cells	
**PD-L1** [14]	Tumor cells	atezolizumab avelumab durvalumab
	Monocytes	
	NK	
	CAFs	
	Macrophages	
	DCs	
	T cells	
**CTLA-4** [13]	T cells	ipilimumab
	NK	
**LAG3** [13,15]	T cells	relatlimab
	NK	
	Plasmacytoid DCs	
	TAMs	
	B Cells	

Commonly used checkpoint molecules and representative types of cells that express these checkpoints are presented in addition to the FDA-approved antibodies available for clinical use (PD-1 [13], PD-L1 [14], CTLA-4 [13], and LAG3 [13,15]). Abbreviations of cell types include: tumor-associated macrophages (TAMs), natural killer cells (NK), dendritic cells (DC), myeloid-derived suppressor cells (MDSCs), and cancer-associated fibroblasts (CAFs).

## 2. Epidemiology

The incidence of CIC depends on the type of ICI used, cancer type, and patient characteristics (Table 2) [7]. Among melanoma patients who were treated with nivolumab plus ipilimumab vs. nivolumab vs. ipilimumab alone, the incidence rate of any grade of diarrhea was 45%, 21%, and 34%, respectively, whereas the incidence of any grade of colitis was 13%, 2%, and 11%, respectively [16]. The incidence of diarrhea was 30.2% to 35.4% for patients treated with CTLA-4 antibodies and 12.1% to 13.7% for patients treated with single-agent anti-PD1 or anti-PD-L1 therapy [17]. For patients treated with anti-CTLA-4 therapy, colitis of any grade was noted in 9.1% of patients, whereas 6.8% experienced grade 3 or 4 colitis [18]. However, with single-agent anti-PD-1 therapy, the incidence of colitis was lower (1.4% experienced any grade of colitis, and 0.9% experienced grade 3 or 4 colitis) [18]. For single-agent anti-PD-L1, 1% of patients experienced colitis and 0.6% experienced grade 3 or 4 colitis [18]. In a metareview, the data were in agreement with the original nivolumab plus ipilimumab trial published in the New England Journal of Medicine [19]. The combination of anti-CTLA-4 and PD-L1 in the metareview was noted to have a higher incidence rate (13.6% of patients experienced any grade of colitis, and 9.4% experienced grade 3 or 4 colitis) [18]. In addition, the general timeline for CIC is different for the ICI agents. With CTLA-4, symptoms typically start manifesting within 5 to 8 weeks, whereas with anti-PD-1 or PD-L1 treatment, colitis typically occurs 3 to 6 months after initiating treatment [7,10,20].

The difference in incidence of colitis/diarrhea was also noted based on the type of cancer. Around 41% of patients with melanoma receiving a CTLA-4 inhibitor alone developed any grade of diarrhea, compared to 25% to 27% patients with lung cancer. With PD-1 or PD-L1 alone, the incidence of diarrhea was 5% to 14% in lung cancer patients in comparison to 10% to 22% in melanoma patients [7]. Patient-specific factors were also noted to contribute to checkpoint inhibitor induced colitis. Use of NSAIDs and vitamin D increases and decreases the incidence of colitis, respectively [31,32]. Patients with a preexisting autoimmune disease, especially IBD, are at increased risk of CIC. For example, patients with underlying IBD who received the antibody against CTLA-4 had a cumulative incidence of CIC of 28% to 30% [17,33,34]. Another note is that the definition of colitis is clinically based. Many patients with diarrhea may only have colitis-type processes when examined with colonoscopy but did not meet the definition for checkpoint inhibitor-induced colitis. 

## 3. Common Terminology Criteria for Adverse Event Grading of CIC

Diarrhea and enterocolitis are two different entities of the same clinical spectrum, where diarrhea is defined as an increased frequency of stool and colitis is defined as abdominal pain, rectal bleeding, or the presence of mucus in the stool with either clinical or radiological evidence of entero-colonic inflammation [2,35]. The National Cancer Institute’s Common Terminology Criteria for Adverse Events (CTCAE; https://ctep.cancer.gov/protocoldevelopment/electronic_applications/ctc.htm; accessed on 21 December 2022) focuses on clinical symptom criteria for autoimmune toxicities such as colitis. The criteria include stool frequency over baseline diarrhea, abdominal pain, blood in stools, color of the blood in the stool, peritoneal signs, and life-threatening colitis criteria, such as colonic perforation, ischemia, necrosis, and toxic megacolon [36,37].

## 4. Inflammatory Bowel Disease

One putative causative mechanism of IBD is the dysfunctional interaction between gut microflora and the mucosal immune system [8]. IBD includes CD and UC, which are differentiated by their location and depth of bowel wall involvement. The incidence and prevalence of IBD is higher in North America/Europe and lower in Asia [38]. Over 1.5 million and 2 million people suffer from CD and UC, respectively, in North America [39]. The incidence in North America ranges from 2.2 to 19.2 cases per 100,000 person-years for UC and 3.1 to 20.1 cases per 200,000 person-years for CD [40].

Three essential factors appear to play a major role in the pathophysiology of IBD: host susceptibility, gut microflora, and mucosal immunity [41]. Host susceptibility factors include genetic alterations, such as the association gene that encodes cytoplasmic protein NOD2 (also known as CARD 15), conferring a higher risk of CD [42]. At this point, it is not clear whether a defect in mucosal immunity leads to an inappropriate immune response against normal gut flora or an altered gut microbiome initiates a pathological response from normal mucosal immune cells [8]. Nonpathogenic bacteria can trigger abnormal immune responses in the presence of defects in the mucosal surface, whereas pathogenic bacteria may mediate low-grade infections that prime the immune response [8,43,44]. In a susceptible patient, loss of self-tolerance leads to IBD via the effector T cells reacting with microbial antigens in the mucosal surface [8]. In CD, type 1 helper T cells are more active and secrete cytokines (tumor necrosis factor α, interferon γ, and interleukin (IL)-12), whereas in UC, a type 2 helper T cell infiltrate predominates in the colon via the activity of transforming growth factor β and IL-5 [42].

CD and UC are also differentiated from each other by the location of the lesion and the extent of bowel wall involvement. CD is usually transmural, involves predominantly the terminal ileum and colon, presents as skip lesions, and frequently leads to fistulas and strictures. UC presents with superficial ulcers beginning from the rectum, extending proximally in the colon, and very rarely results in strictures or fistulas [38]. The clinical presentation of IBD can include fever, abdominal pain, diarrhea, blood in the stool, weight loss, and perianal disease (fissure or fistula) [45]. Though there is no gold standard of diagnosis for IBD, the clinical scenario, biochemical studies, radiographic imaging, and endoscopy with histopathology guide the physician into the spectrum of diseases that include IBD [38,46]. The histopathology of UC and CD is associated with distinct changes in crypt architecture, inflammatory cell infiltration, and epithelial metaplasia [38,47]. In UC, these changes are continuous and there is no granuloma formation, but in CD, findings are patchy, with a cobblestone appearance and rectal sparing with granuloma formation [38,47].

Different scoring systems have been developed to assess the severity of IBD. The Crohn’s Disease Activity Index (CDAI) is the most widely used scoring system for CD. The CDAI uses the number of stools, abdominal pain, general wellbeing, extraintestinal complications, antidiarrheal agent use within the last 7 days, abdominal mass felt on palpation, hematocrit, and body weight to calculate the score. Scores range from 0 to 600, where <150 corresponds to remission, 150 to 219 denotes mildly active disease, 220 to 450 signifies moderately active disease, and >450 depicts severe disease [48]. The Crohn’s Disease Endoscopic Index of Severity (CEDIS) is the first scoring system that incorporated endoscopic findings in the scoring method. Scores range from 0 to 44, with higher scores indicating more severe disease [49]. The most widely used scoring system for UC is the Mayo Score/Disease Activity Index. There are four parameters scored on a scale of 0 to 3 (normal, mild, moderate, severe): stool frequency (normal, >1–2 than normal, >3–4 than normal, or >4 than normal), rectal bleeding (none, visible blood < 50% bowel movement, visible blood > 50%, or only blood with no stool), endoscopic mucosal appearance, and physician’s rating of disease control [50]. The highest score possible is 12.

The treatment goals of IBD are to facilitate clinical remission and achieve steroid sparing for long-term maintenance [38]. Corticosteroids are very effective in acute flares and to induce remissions. However, long-term use is plagued with side effects, including hyperglycemia, moon face, acne, mood disturbance, osteoporosis, and cataracts [51]. The first-line therapy in mild to moderate UC is oral 5-aminosalicylate (5-ASA) [38,52,53,54], but 5-ASA is not effective for CD [55]. Immunomodulator agents, such as azathioprine/6-mercaptopurine (6-MP), are usually efficacious for both CD and moderate to severe UC [56,57]. Antibodies targeting TNFα (infliximab, adalimumab, golimumab, and etanercept) represent a major advancement for the treatment of IBD [58,59]. Other biologics, such as anti-integrin antibodies (vedolizumab) and anti-IL-12/23 antibodies (ustekinumab), were found to be efficacious for patients who could not tolerate TNFα-directed antibodies or became refractory to TNFα treatment. In one trial, tofacitinib, a JAK2 inhibitor, was found to be more effective for induction and maintenance than the placebo in moderate to severe UC and is FDA-approved for patients with UC who are refractory to antibody therapy or experience intolerable toxicity to these agents [60]. Additional agents are currently in development, including modulate lymphocyte trafficking (e.g., anti-α4β7 integrin antibodies) and agents that inhibit either SMAD 7 (mongersen) or phosphodiesterase 4 [38,41,42]. 

Symptomatic relief, endoscopic remission, and histological response are three possible therapeutic outcomes for IBD [61]. The decision of which endpoint to use is not well-established. According to the British Society of Gastroenterology, consensus suggests that symptoms’ resolution and mucosal healing are the preferred treatment outcomes of IBD [54]. Symptomatic relief in a patient is reported as improvement in symptoms, such as diarrhea and abdominal pain. Endoscopic remission is defined as a lack of findings, with absence of visible blood, ulcer, erosion, or erythema, and definitive evidence of mucosal healing on endoscopic evaluation [61]. Lichtenstein et al. defined histological remission for UC as both macroscopic and microscopic healing with a UC Disease Activity Index score of 0 [62]. However, the presence of skip lesions and lack of a validated endoscopic scoring system make identification of histological remission difficult in CD [63]. Presence of neutrophils, eosinophils, lymphoid follicles, or plasma cells in lamina propria, erosion, and cryptitis are considered markers of inflammation. In real-world practice, most clinicians aim for clinical remission that includes patient-reported outcomes and endoscopic healing. However, histological remission has been associated with a lower relapse rate, lower rates of hospitalization, and a lower risk of colon cancer [64,65,66]. Due to the lack of well-defined criteria with newer therapies, including biologic agents, histological remission is a goal to aspire to but is not frequently used in routine clinical practice. In CIC, the role of histological remission is not well-studied. However, increasingly, colonoscopy is being used to diagnose CIC and it is likely that we will have data on this phenomenon in the next few years [5,67].

## 5. CIC Similarities to IBD

ICIs remove the inhibitory signals on T cells, macrophages, tumor cells, and other cell types to permit the immune system to attack the cancer [68]. A similar mechanism is involved in the pathogenesis of IBD, where altered gut microbiota leads to dysregulation of effector T cells and regulatory T cells. Dendritic cells present bacterial antigens to naïve T cells in the gut mucosa, and those naïve T cells differentiate to effector, regulatory, and memory cells. Effector T cells produce interferon-γ and recruit macrophages and CD8^+^ T cells. Regulatory T cells express the immune checkpoint molecule CTLA-4 and produce anti-inflammatory cytokines, such as IL-10 and TGF-β, to blunt the immune response. Dysregulation of effector and regulatory T cells results in overstimulation of the immune system in the gut mucosa and IBD [69].

Several studies have compared features (physical exams, histology with immune architecture, and endoscopic findings) of CIC to IBD [9,10,11,12]. In general, CIC may clinically present as either acute or chronic colitis (CD, UC, or chronic lymphocytic colitis). Similar to IBD, mucosal immune responses predominate in the pathology [10]. CTLA-4 is expressed on T cells, is located on chromosome 2q33, and maintains immune tolerance by interacting with the B7 family of molecules on the surface of antigen-presenting cells (APCs). One of the variants of CTLA-4 is +49, which causes a threonine-to-alanine amino acid substitution in position 17 and leads to a reduction of its cell surface expression. Another variant of CTLA-4, CT60, is associated with CTLA-4 functional expression [70]. Both of these variants, especially CTLA-4 CT60, are associated with autoimmune diseases, such as type 1 diabetes mellitus, celiac disease, and autoimmune thyroid disease [71].

Another meta-analysis demonstrated an association of the CTLA-4 +49A/G variant and CD in Caucasian populations, whereas the association of the CTLA-4 CT60 variant and UC was seen in Asian populations [70]. It is approximated that 59% of patients with heterogenous germline mutations of CTLA-4 presented with gastrointestinal manifestations, such as diarrhea with malnutrition, pancreatic insufficiency, atrophic gastritis, CD, and celiac disease [72]. Similarly, there is an association between defective PD-1/PD-L1 signaling pathways and IBD [73]. In murine models, a mechanism of upregulation of PD-L1 when exposed to food particles and bacterial flora was elucidated [74]. Calcium and other ions form nanoparticles with digested proteins and bacterial peptidoglycans. This complex migrates into the Peyer’s patches, where it interacts with APCs. APCs then express PD-L1 to control excessive immunity to food particles. In CD, APCs fail to express PD-L1 [53]. 

The clinical presentation of CIC is often similar to IBD in that endoscopic findings of CIC include mucosal ulceration or nonulcerative inflammation, including erythema, exudate, edema, friability, or normal appearance, such as is seen in microscopic colitis [12,75]. Mucosal ulceration of CIC can be deeply patchy, as seen in CD, or express continuous lesions in the mucosa, as seen in UC [10]. Most CIC involves the left colon, with rectal sparing [10,12]. The histology can be categorized as acute, chronic, or normal. The acute pattern of CIC typically demonstrates neutrophilic infiltrate, cryptitis, crypt abscess, and apoptosis, whereas the chronic pattern is defined by basal lymphocytic infiltrate, distortion of cryptic architecture, and Paneth cell metaplasia [12]. Unlike IBD, cryptic branching, basal layer plasmacytosis, and increase in lamina propria plasma cells are minimal in CIC [11]. Immunophenotypic analysis also revealed infiltration of CD4^+^ cells in lamina propria in CIC in addition to high levels of mucosal interferon-γ and IL-17A [10]. The distribution of CD8^+^ T cells, CD4^+^ T cells, and CD68^+^ cells within the mucosa in the patients with CIC has more similarities with UC than CD [76,77]. These findings suggest that CIC has similarity to IBD in terms of pathophysiology, symptoms, site of involvement, and immune cell infiltrate.

Given the common immunophenotypic etiologies of CIC and IBD, therapies are also closely related. For grade 1 toxicities, close monitoring and supportive management are recommended. For grade 2 toxicities, the ICI is typically held with the use of a steroid taper. Close observation is recommended until the symptoms revert to grade 1 or less. For grade 3 toxicities, a high-dose corticosteroid taper is initiated over 4 to 6 weeks. Additional management includes the use of prophylaxis for pneumocystis pneumonia, shingles and upper gastrointestinal prophylaxis with twice daily H_2_ blockers, depending on the clinical scenario, and is fully described in the National Cancer Comprehensive Network (NCCN) and Society for Immunotherapy of Cancer (SITC) guidelines [37,78]. If the patient does not respond to steroids, then anti-TNFα agents, such as infliximab or the anti-integrin antibody vedolizumab, are used and consultation with gastroenterologists is recommended. Although it is difficult to properly collect, fecal calprotectin (S100A8/A9) can be measured serially to monitor the change in the level of inflammation [79]. In addition, colonoscopy is typically considered with gastrointestinal consultation. Furthermore, with grade 3 colitis, the therapy responsible for the colitis is typically discontinued and the patient is monitored for signs of cancer progression [78]. For grade 4 toxicities, permanent cessation of the ICI is recommended [16,78].

## 6. Role of Diets Recommended for IBD in Prevention and Treatment of CIC

It is postulated that, in a genetically susceptible patient, altered gut microflora results in chronic inflammation, ultimately leading to IBD [80]. Maintaining gut microbiota and optimizing nutritional absorption through the diet is an important strategy for achieving or maintaining remission in IBD and may also be useful for CIC. Several diets and nutritional supplements have been studied for the management of acute flares and long-term management of IBD. Here, we will discuss the role of different diets in IBD and their potential benefits in CIC (Table 3).

### 6.1. Exclusive Enteral Nutrition for Active CD

This first dietary study for CD performed in 1971 demonstrated symptomatic improvement, including reducing abdominal pain and weight gain, in hospitalized patients with severe CD [81]. Voitk et al. described a formula consisting of hydrolysate, synthetic amino acid, digestible fat, sucrose, vitamins, and minerals dissolved in water, which was delivered via a nasogastric tube [81]. Exclusive enteral nutrition (EEN) is an elemental, semi-elemental, and defined formula recommended for actively hospitalized CD patients. This diet is proposed to change the gut microbiota profile and promote mucosal healing [82]. EEN was shown to be as effective as corticosteroids in children with severe CD [83], but this was not the case in adults. Comparing the effect of enteral nutrition with liquid oligopeptides as a sole therapy to steroid and sulfasalazine combination in adults with active CD showed that 52% (n = 29/55) of patients achieved remission in 30.7 days with the enteral diet and 78% (n = 41/52) of patients in the steroid group achieved remission in 8 days [84]. Though this study compared diet vs. sulfasalazine with steroids, diet alone was found to be less effective than steroids, but there were no significant side effects in the diet group compared to the medication group. However, use of enteral nutrition with oligopeptides had difficulty sustaining long-term remission and lacked palatability [85]. It is common clinic practice, and we also endorse that steroids should be used first to control symptoms for grade 3 or 4 colitis with bowel rest. A form of an EEN diet should be considered as the first test diet after symptoms have been controlled with steroids in preparation for transition to a diet more fitting for the patient. There are modern equivalents that are commercially available. Patients also need to be aware that they may not be able to resume their pre-checkpoint inhibitor diets.

### 6.2. Banana, Rice, Applesauce, and Toast (BRAT)

The BRAT diet includes bananas, rice, applesauce, and toast. It is usually recommended for acute gastroenteritis but is also used during an active flare of IBD. These are easily digestible and more easily tolerated foods that are low in fiber, protein, and fat, but it significantly lacks nutritional elements such as protein, fiber, calcium, vitamin D, and vitamin B12; therefore, it should not be continued for a long period of time. In 1990, the American Academy of Pediatrics eliminated this diet from their guidelines [86]. It can be used initially when managing grade 2 colitis or when transitioning to a solid diet during treatment of grade 3 CIC, but given the nutritional deficiencies induced with this diet, we would not recommend this diet for long-term use in CIC patients.

### 6.3. Specific Carbohydrate Diet (SCD)

The specific carbohydrate diet (SCD) was first described in 1924 for celiac disease and was later popularized for IBD by biochemist Elaine Gottschall through her book “Breaking the Vicious Cycle” [94]. The SCD excludes complex carbohydrates such as grains and dairy and includes monosaccharides such as fresh fruits, fresh vegetables, and honey [94]. It is believed that disaccharides and polysaccharides, such as refined sugar, lactose, and starch, causes injury to small intestinal surfaces. This process impairs digestion of disaccharides and leads to bacterial overgrowth and inflammation [94]. The SCD demonstrated promising results in pediatric studies, with significant clinical improvement. The study showed that in children with active CD, the pediatric CD activity index dropped from 32.8 ± 13.2 at baseline to 20.8 ± 16.6 by 4 ± 2 weeks and to 8.8 ± 8.5 by 6 months [96]. Recently, the SCD was compared to a Mediterranean diet in adults with CD, and SCD was not proven to be superior to the Mediterranean diet in terms of achieving symptomatic remission or in reducing fecal calprotectin or C-reactive protein in blood [97]. Therefore, once a patient is able to tolerate a Mediterranean diet, the SCD diet may not be superior, but it can be useful when patients do not eat fish-based products. 

### 6.4. Low Fermentable Oligosaccharides, Disaccharides, Monosaccharides, and Polyols (FODMAP) Diet

The low fermentable oligosaccharides, disaccharides, monosaccharides, and polyols (FODMAP) diet was initially described for irritable bowel syndrome. Highly fermentable short-chain fatty acids are poorly absorbed and increase gut permeability in the distal small bowel and proximal large intestine [90]. The FODMAP diet includes fruits, corn syrup, milk, yogurt, wheat, onions, apple, pears, legumes, beans, and other similar products. In a recent study, there was a significant increase in the number of patients reporting satisfactory symptoms’ relief between baseline (n = 14/88 (16%)) and those following a low FODMAP diet (n = 69/88 (78%); *p* < 0.001) for functional gastrointestinal symptoms, such as chronic abdominal pain, flatulence, and diarrhea, without any acute inflammation [91]. 

The concept of the FODMAP diet overlaps with the SCD. The SCD has no restriction for fruits and vegetables, whereas the FODMAP diet has restrictions for certain fruits and vegetables [98]. The FODMAP diet allows starch, such as gluten-free bread or cereal products, rice, quinoa, or oats, which are strictly prohibited in the SCD. The FODMAP diet also avoids excess fructose products, such as those seen in fruits, juices, or sweeteners. Vegetables such as asparagus, broccoli, cabbage, eggplant, garlic, and onion are also restricted in the FODMAP diet, but the SCD does not restrict fruits or vegetables. One potential advantage of the FODMAP diet is that it does not include fructose-containing products, which may cause intestinal inflammation in murine studies [99,100]. The SCD requires long-term adherence, whereas the FODMAP diet can be used in the short term and is less restrictive than the SCD. For these reasons, we believe the FODMAP diet is more relevant to CIC patients while they slowly recover from CIC symptoms. 

### 6.5. Semi-Vegetarian Diet and Whole Food Plant-Based Diet

In Western countries, it is proposed that the incidence of IBD is increasing due to the intake of a diet rich in refined carbohydrates/animal fats/proteins and low in fruits/vegetables [90,92]. The Western diet is believed to increase proinflammatory cytokines, increase intestinal permeability, and alter the microbiome environment, causing chronic inflammation [80]. In Japan, a plant-based semi-vegetarian diet (SVD) was compared to an omnivorous diet to prevent relapse of IBD. The SVD included mostly fresh fruits and vegetables, with weekly fish and biweekly meat [92]. Among 16 patients (73% compliance) who maintained the SVD, CD remission was maintained in 94%, whereas the remission rate of CD was 33% among patients following the omnivorous diet [92]. In 2018, a similar study was conducted on UC patients in two tertiary care centers in Japan. It was a single-arm study in which patients with UC who did not need immediate treatment were admitted for 2 weeks and an SVD was strictly administered. Cumulative relapse rates at 1, 2, 3, 4, and 5 years during follow-up after educational hospitalization were 2%, 4%, 7%, 19%, and 19%. However, 77% of the patients illustrated improvement of symptoms, including reductions in the amount and frequency of bloody stool during hospitalization [101].

The whole food plant-based diet includes only fresh fruits, vegetables, and whole-grain foods without any animal product, including dairy, eggs, food emulsifiers, artificial flavors, omega 6 fatty acid, and any processed food [95]. Efficacy has only been reported in one case report. No prospective studies are available. The SVD or whole food plant-based diets can also be considered for patients with CIC with grade 2 colitis or in the transition period from the acute phase of CIC to a full diet. 

### 6.6. Anti-Inflammatory Diet/Diet on Autoimmune Protocol

This diet avoids gluten and refined sugars. It has an initial 6-week elimination phase, followed by a 4- to 5-week maintenance phase. In the elimination phase, the patient avoids grains, legumes, dairy, eggs, coffee, alcohol, nuts, seeds, refined carbohydrates, oils, and food additives. These are the foods believed to cause inflammation and dysbiosis. It focuses on consuming fresh, nutrient-dense foods such as fruits, freshly cooked vegetables, fish or meat, bone broth, and fermented food. Then, in the maintenance phase, the patient remains on the diet to see the results. In the reintroduction phase, patients gradually introduce food groups to ascertain which foods aggravate their symptoms and to determine a personalized sustainable diet [89]. This diet and phasic reintroduction of food can be considered after CIC patients recover from the acute phase, but additional data would be needed to promote long-term use.

### 6.7. Mediterranean Diet

The Mediterranean diet consists of vegetables, fruits, cereals, nuts, legumes, unsaturated fats (e.g., olive oil), a medium intake of fish, dairy products, wine, and low consumption of saturated fats, meat, and sweets [93]. The Mediterranean diet contains a large volume of soluble fiber, which is proposed to maintain intestinal eubiosis [80]. The diet can promote diverse microbiota, which can result in normalization of symptoms in IBD patients [102]. In one study of 142 patients with IBD, increases in fecal short-chain fatty acids promoted the activity of regulatory T cells in the gut and increased expansion of *Lacnospira* and *L. ruminococcus* bacterial species that are usually reduced in IBD [93]. Interestingly, a randomized control trial was conducted comparing the effectiveness of the SCD and the Mediterranean diet in mild to moderate CD, and it did not demonstrate any significant differences in symptom control between these types of diets [97]. For CIC patients, the Mediterranean diet can be studied further to show whether to recommend it at ICI initiation to maintain intestinal eubiosis and prevent development of inflammation in the first place.

### 6.8. Low-Residue Diet

The low-residue diet focuses on the reduction of “residue” or undigested food in the stool. This diet decreases high-fiber foods, such as whole-grain breads, cereals, nuts, fruits, and vegetables. This is carefully distinguished from a low-fiber diet, which only eliminates insoluble fiber. Some CD patients have experienced an improvement in their obstructive symptoms and strictures with this diet [87]. Specifically, the low-fiber diet promotes a local environment suitable for expansion of adherent-invasive *Escherichia coli* (AIEC), a known species that is associated with CD pathology [88]. We would recommend a low-residue diet once patients have recovered from the acute phase of high-grade (grade 3–4) colitis and are resuming oral intake. The low-residue diet can also be recommended to patients with CIC after resolution of the acute phase of colitis to help sustain remission. However, patients with CIC do not often have strictures such as those experienced by CD patients, and therefore, the low-residue diet may not have the same utility.

### 6.9. Dietary Supplements

In addition to dietary modification, dietary supplements are sometimes necessary due to poor absorption in IBD patients. Absorption of essential nutrients, such as fat-soluble vitamins and vitamin B12 through the terminal ileum, can be significantly impaired in IBD [103].

Vitamin D plays an important role in innate immunity, maintaining gut wall integrity, and in the development and function of T cells [104,105]. The polymorphism of the vitamin D receptor (VDR) has been associated with increased susceptibility to IBD in Ashkenazi Jewish populations in one Israeli study [106]. In preclinical murine models, deficiency of vitamin D increases the severity of dextran sodium sulfate-induced colitis [107]. Li et al. performed a meta-analysis including 18 randomized controlled trials involving 908 patients with confirmed diagnoses of either UC or CD. The 25(OH) vitamin D3 level, relapse rate, inflammation index, and adverse events were compared between patients receiving vitamin D supplementation and control groups receiving either a placebo or low-dose vitamin D. The result of the meta-analysis demonstrated that vitamin D can reduce the relapse rate of IBD by 64%, but there was no benefit to excessive amounts of vitamin D supplementation [108]. Interestingly, a retrospective study of 213 patients with melanoma receiving over-the-counter vitamin D supplementation showed a reduced rate of colitis in patients receiving anti-PD-1, anti-CTLA-4, or combination immune-based therapy [32].

Supplementation of omega 3 fatty acid is debatable. In one study, N3 polyunsaturated fatty acid was found to be in higher abundance than the inflammatory N6 polyunsaturated fatty acid in patients who were in remission from UC and CD [109]. However, further analysis with supplementation of fish oil in both UC and CD found this regimen to not be effective [110,111,112]. 

Curcumin is a polyphenol derivative from turmeric plant that has anti-inflammatory and antioxidant properties. One putative mechanism is that curcumin inhibits the NF-kB inflammatory pathway [113]. A multicenter, randomized, placebo-controlled, double-blinded study of 50 patients with mild to moderate UC receiving oral or rectal mesalamine demonstrated that 53.8% of patients who received curcumin with mesalamine achieved remission at week 4, whereas none of the patients in the placebo group receiving mesalamine alone achieved remission [114]. 

Considering the conflicting data with numerous vitamin supplementations at high doses, our general approach is to replete only those vitamins that are deficient in the patient in the setting of CIC. Nutritional supplements do have medicinal value, but they should be studied in the clinical setting prior to widespread use. We tend to avoid mega-doses of nutritional supplements in this setting unless closely monitored under the context of a clinical trial, given multiple reports of nutritional supplements acting as anti-cancer and pro-cancer agents and unknown interactions with our standard regimens. 

## 7. Role of Diet Changes in Microbiota

The roles of diets and changes in the microbiota treatment response have been studied for IBD (Table 4). In rural Africa where IBD is rare, people consume an agrarian diet that is enriched with high fiber. This is associated with an increase in *Prevotella* species in the microbiota. *Prevotella* ferment dietary fiber and produce short-chain fatty acid-like butyrate, propionate, and acetate that maintain a healthy mucosa and production of anti-inflammatory interleukins [80,98,115]. However, a diet rich in animal protein/fat and refined sugar produces a microbiota environment rich in *Bacteroides* species. These bacterial species increase gut wall permeability, leading to inflammation [80,115]. Animal studies confirm these findings. Mice were fed either a high-fat diet or a normal diet and then exposed to dextran sodium sulfate (DSS) to induce colitis. Mice receiving a high-fat diet demonstrated an increased incidence of DSS-associated colitis, with higher frequency of natural killer T cells infiltrating colonic tissue associated with upregulation of TNFβ and interferon-γ [116]. The intake of an omnivorous diet, including multiple animal products, in IBD, and specifically in CD, is associated with inflammation and direct toxic effects, including a loss of goblet cells, crypt architectural distortion, and superficial mucosal ulceration [98,117].

## 8. Role of the Microbiome

The role of diet and the microbiome has been extensively studied for enhancing responses to ICIs and may have significance for CIC. The T cell response to a CTLA-4 inhibitor was associated with *Bacteroides* species in murine studies [123]. *Bifidobacterium* species enhances CD8^+^ T cell tumor infiltration [124]. In a recent observational study of 128 melanoma patients, it was found that patients who ingested a high-fiber diet were more likely to respond to anti-PD-1 therapy in terms of PFS than people who ingested a low-fiber diet (hazard ratio, 0.71 (95% CI, (0.52, 0.98)). The data suggested that this effect may be mediated by increased T cell activation and accumulation of both CD4^+^ and CD8^+^ T cells within the melanoma microenvironment [118]. Considering the relationship between the gut microbiome and ICI responses along with modulation of microbiota by diet, it is reasonable to postulate that diets favorable for a response in IBD may also have benefit in CIC and improve responses to ICIs (Table 5). It is the hope that this review and clinical perspective will serve as the catalyst for more studies in this area. 

## 9. Conclusions

**Figure 1:**
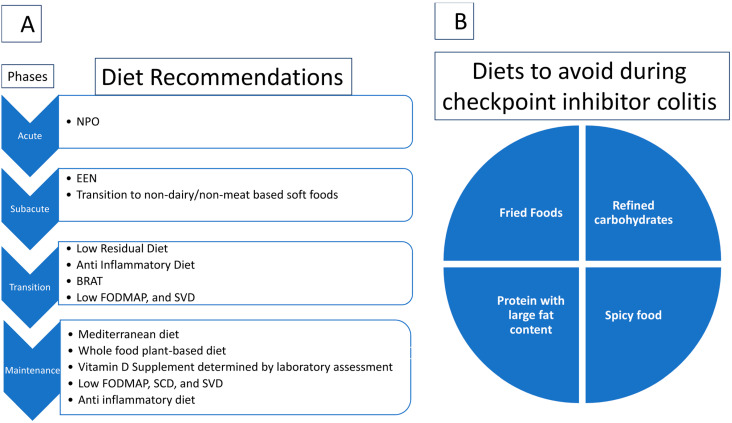
(**A**) Diet recommendations per stage. Abbreviations: BRAT, banana, rice, applesauce, and toast; EEN, exclusive enteral nutrition; Low FODMAP, low fermentable oligosaccharides, disaccharides, monosaccharides, and polyols; NPO, nothing by mouth; SCD, specific carbohydrate diet; SVD, semi-vegetarian diet. A diet is typically introduced after acute colitis symptoms have subsided with high-dose steroids during the transition period. (**B**) Diets to avoid during checkpoint inhibitor colitis toxicity. Large meals should be avoided. In addition, fried foods, refined carbohydrates, proteins with large fat contents, and spices (red hot peppers, chili peppers, etc.) should also be avoided.

Implementation of IBD dietary recommendations in patients receiving ICI treatment may have significant impacts on reducing the incidence and severity of colitis (Figure 1, Table 3).

Further studies are encouraged to show direct associations of these dietary changes with CIC. From the current literature, several conclusions are possible. First, when patients present with active symptoms associated with grade 3 or 4 CIC, strict gastrointestinal rest and steroids are initiated. A diet is typically introduced after acute colitis symptoms have subsided with high-dose steroids during the transition period. In current practice, first we try a clear liquid diet including jello without any fat or fiber in it. If patients tolerate this, exclusive enteral nutrition consisting of liquid formulations are typically the first diet recommendations. The FODMAP diet or the SVD in the absence of food groups high in residues (e.g., seeds and nuts) are good options for the period of transitioning to solid food. The specific carbohydrate diet, Mediterranean Diet, plant-based diet, anti-inflammatory diet, and low-residue diet are suited for the chronic phases while transitioning to a full diet tolerated by the patient. In all cases, it is recommended to have a bland diet. Further studies are needed to consider specific diets when initiating checkpoint blockade to prevent checkpoint-induced colitis. It is reasonable at this point to suggest a Mediterranean diet and check for vitamin D deficiency.

## Figures and Tables

**Table 2:** Commonly used checkpoint antibodies in cutaneous malignancies with incidence of diarrhea and colitis.

Type of Cutaneous Malignancy	Immunotherapy	Diarrhea		Colitis	
		Grade ≤ 2	Grade ≥ 3	Grade ≤ 2	Grade ≥ 3
**Melanoma**	Pembrolizumab (Keynote-054) [21] *	18.3%	0.8%	1.7%	2%
	Nivolumab (Checkmate-238) [22] *	22.8%	1.5%	1.3%	0.7%
	Ipilimumab (EORTC-18071) [23]	39%	10%	8%	7%
	Ipilimumab (3 mg/kg) + Nivo (1 mg/kg) (Checkmate-511) [24] *	24.7%	6.2%	0.6%	4.5%
	Ipilimumab (1 mg/kg) + Nivolumab (3 mg/kg) (Checkmate-511) [24] *	23.3%	2.8%	1.7%	2.2%
	Nivolumab + Relatlimab (Relativity-047) [25] *	12.7%	0.8%	5.7%	1.1%
**SCC**	Pembrolizumab (Keynote-629) [26] *	9.4%	0%	0%	1.3%
	Cemiplimab [27]	27%	0%	-*	-*
**BCC**	Cemiplimab [28]	24%	0%	0%	5%
**MCC**	Pembrolizumab (Keynote 017) [29] *	3.8%	0%	-*	-*
	Avelumab [30]	5.1%	0%	-*	-*

Commonly used checkpoint antibodies in cutaneous malignancies with incidence of diarrhea and colitis. In melanoma, these antibodies include: pembrolizumab [21], nivolumab [22], ipilimumab [23], ipilimumab + nivolumab [24], and nivolumab and relatlimab [25]. In squamous cell carcinoma (SCC), these antibodies include: pembrolizumab [26] and cemiplimab [27]. In basal cell carcinoma (BCC), cemiplimab is utilized [28]. In Merkel cell carcinoma (MCC), these antibodies include: pembrolizumab [29] and avelumab [30]. * Grade 1–2 is calculated from incidence of all grade and grade 3 above diarrhea and colitis. -* Colitis was not specifically tabulated in these studies. It is an important clinical distinction between diarrhea and colitis, and it is the goal of this review to bring attention to this matter.

**Table 3:** Dietary recommendations in IBD.

Diet	Contents	Use in CIC
Exclusive enteral nutrition [81,82,83,84,85]	Formula consisting of hydrolysate, synthetic amino acid, digestible fat, sucrose, vitamin, mineral dissolved in water	Subacute
BRAT [86]	Banana, rice, applesauce, and toast	Transition
Low-Residual Diet [87,88]	Excludes high-fiber foods, such as whole-grain breads, cereals, nuts, fruits, and vegetables.	Transition
Anti-Inflammatory Diet [89]	Includes fresh nutrient-dense food such as fruits, freshly cooked vegetables, fish or meat, bone broth, and fermented food	Transition/Maintenance
Low FODMAP [90,91]	Excludes foods such as fruits, corn syrup, milk, yogurt, honey, wheat, onions, apple, pears, legumes, beans, and other similar products.	Transition/Maintenance
Semi-Vegetarian Diet [92]	Includes mostly fresh fruits and vegetables with weekly fish and biweekly meat	Transition/Maintenance
Mediterranean Diet [93]	Includes vegetables, fruits, cereals, nuts, legumes, unsaturated fat (e.g., olive oil), a medium intake of fish, dairy products, wine, and a low consumption of saturated fat, meat, and sweets	Maintenance
Specific Carbohydrate Diet [94]	Includes monosaccharides such as fresh fruits, fresh vegetables, honey, meat, and fish. Excludes complex carbohydrates such as grains and dairy.	Maintenance
Whole food plant-based diet [95]	Includes fresh fruits, vegetables, and whole grain foods without any animal product, including dairy, eggs, food emulsifiers, artificial flavors, omega 6 fatty acid, and any processed food	Maintenance

**Table 4:** Impact of diet on gut microbiota.

Diet	Abundance of Bacterial Species
High-Fiber Diet [80,115,118]	Ruminococcaceae species, Prevotella, Firmicutes, Proteobacteria
Meditarranean Diet [93,119]	Lacnospira, L. ruminococcus species, Bacteroidetes, Clostridium cluster IV, and XIVa
Specific Carbohydrate Diet [119,120]	Enterobacter species, including Escherichia, Clostridia, Gammaproteobacteria
Low FODMAP Diet [119,121]	Bacteroides, Clostridium cluster XIVa, Akkermansia muciniphilia
Whole Food Plant-Based Diet [122]	Prevotella, Ruminococcus

Impact of diet on gut microbiota. The abundance of bacterial species with the high-fiber diet [80,115,118], Mediterranean diet [93,119], specific carbohydrate diet [119,120], low FODMAP diet [119,121], and whole food plant-based diet [122] are presented.

**Table 5:** Effect of microbiota species on checkpoint inhibitors.

Bacterial Species	Effect of Immunotherapy
Bacteroides fragillis, Bacteroides thetaiotaomicron, and Burkholderiales	Increase efficacy of CTLA-4 inhibitors [123]
Bifidobacterium breve, Bifidobacterium longum, and Bifidobacterium adolescentis	Augment effect of anti-PD-L1 inhibitors [124]
Ruminococcaceae species and Faecalibacterium species	High-fiber diet increases efficacy of anti-PD-1 therapy [118,125]

Effect of microbiota species on efficacy of checkpoint inhibitors. Associations between bacterial species and efficacy of ICIs, such as CTLA-4 inhibitors [123], anti-PD-L1 antibody [124], and anti PD-1 antibody [118,125], are presented.

## Data Availability

Not applicable.

## References

[1] Goodman A.M., Piccioni D., Kato S., Boichard A., Wang H.-Y., Frampton G., Lippman S.M., Connelly C., Fabrizio D., Miller V. (2018). Prevalence of PDL1 Amplification and Preliminary Response to Immune Checkpoint Blockade in Solid Tumors. JAMA Oncol..

[2] Postow M.A. (2015). Managing Immune Checkpoint-Blocking Antibody Side Effects. Am. Soc. Clin. Oncol. Educ. Book.

[3] Dine J., Gordon R., Shames Y., Kasler M.K., Barton-Burke M. (2017). Immune Checkpoint Inhibitors: An Innovation in Immunotherapy for the Treatment and Management of Patients with Cancer. Asia-Pac. J. Oncol. Nurs..

[4] Carlino M.S., Larkin J., Long G.V. (2021). Immune checkpoint inhibitors in melanoma. Lancet.

[5] Hashash J.G., Francis F.F., Farraye F.A. (2021). Diagnosis and Management of Immune Checkpoint Inhibitor Colitis. Gastroenterol. Hepatol..

[6] Dougan M., Wang Y., Rubio-Tapia A., Lim J.K. (2021). AGA Clinical Practice Update on Diagnosis and Management of Immune Checkpoint Inhibitor Colitis and Hepatitis: Expert Review. Gastroenterology.

[7] Gong Z., Wang Y. (2020). Immune Checkpoint Inhibitor-Mediated Diarrhea and Colitis: A Clinical Review. JCO Oncol. Pract..

[8] Strober W., Fuss I., Mannon P. (2007). The fundamental basis of inflammatory bowel disease. J. Clin. Investig..

[9] Bertha M., Bellaguara E., Kuzel T., Hanauer S. (2017). Checkpoint Inhibitor-Induced Colitis: A New Type of Inflammatory Bowel Disease?. ACG Case Rep. J..

[10] Siakavellas S.I., Bamias G. (2018). Checkpoint inhibitor colitis: A new model of inflammatory bowel disease?. Curr. Opin. Gastroenterol..

[11] Lo Y.C., Price C., Blenman K., Patil P., Zhang X., Robert M.E. (2021). Checkpoint Inhibitor Colitis Shows Drug-Specific Differences in Immune Cell Reaction That Overlap With Inflammatory Bowel Disease and Predict Response to Colitis Therapy. Am. J. Clin. Pathol..

[12] Wang Y., Abu-Sbeih H., Mao E., Ali N., Qiao W., Trinh V.A., Zobniw C., Johnson D.H., Samdani R., Lum P. (2018). Endoscopic and Histologic Features of Immune Checkpoint Inhibitor-Related Colitis. Inflamm. Bowel Dis..

[13] Liu X., Hogg G.D., DeNardo D.G. (2021). Rethinking immune checkpoint blockade: ‘Beyond the T cell’. J. Immunother. Cancer.

[14] Yi M., Niu M., Xu L., Luo S., Wu K. (2021). Regulation of PD-L1 expression in the tumor microenvironment. J. Hematol. Oncol..

[15] Graydon C.G., Mohideen S., Fowke K.R. (2020). LAG3’s Enigmatic Mechanism of Action. Front. Immunol..

[16] Brahmer J.R., Lacchetti C., Schneider B.J., Atkins M.B., Brassil K.J., Caterino J.M., Chau I., Ernstoff M.S., Gardner J.M., Ginex P. (2018). Management of Immune-Related Adverse Events in Patients Treated With Immune Checkpoint Inhibitor Therapy: American Society of Clinical Oncology Clinical Practice Guideline. J. Clin. Oncol..

[17] Tang L., Wang J., Lin N., Zhou Y., He W., Liu J., Ma X. (2021). Immune Checkpoint Inhibitor-Associated Colitis: From Mechanism to Management. Front. Immunol..

[18] Wang D.Y., Ye F., Zhao S., Johnson D.B. (2017). Incidence of immune checkpoint inhibitor-related colitis in solid tumor patients: A systematic review and meta-analysis. OncoImmunology.

[19] Wolchok J.D., Chiarion-Sileni V., Gonzalez R., Rutkowski P., Grob J.-J., Cowey C.L., Lao C.D., Wagstaff J., Schadendorf D., Ferrucci P.F. (2017). Overall Survival with Combined Nivolumab and Ipilimumab in Advanced Melanoma. N. Engl. J. Med..

[20] Weber J., Kähler K., Hauschild A. (2012). Management of Immune-Related Adverse Events and Kinetics of Response With Ipilimumab. J. Clin. Oncol. (JCO).

[21] Eggermont A.M.M., Blank C.U., Mandala M., Long G.V., Atkinson V., Dalle S., Haydon A., Lichinitser M., Khattak A., Carlino M.S. (2018). Adjuvant Pembrolizumab versus Placebo in Resected Stage III Melanoma. N. Engl. J. Med..

[22] Weber J., Mandala M., Del Vecchio M., Gogas H.J., Arance A.M., Cowey C.L., Dalle S., Schenker M., Chiarion-Sileni V., Marquez-Rodas I. (2017). Adjuvant Nivolumab versus Ipilimumab in Resected Stage III or IV Melanoma. N. Engl. J. Med..

[23] Eggermont A.M.M., Chiarion-Sileni V., Grob J.-J., Dummer R., Wolchok J.D., Schmidt H., Hamid O., Robert C., Ascierto P.A., Richards J.M. (2015). Adjuvant ipilimumab versus placebo after complete resection of high-risk stage III melanoma (EORTC 18071): A randomised, double-blind, phase 3 trial. Lancet Oncol..

[24] Lebbé C., Meyer N., Mortier L., Marquez-Rodas I., Robert C., Rutkowski P., Menzies A.M., Eigentler T., Ascierto P.A., Smylie M. (2019). Evaluation of Two Dosing Regimens for Nivolumab in Combination With Ipilimumab in Patients With Advanced Melanoma: Results From the Phase IIIb/IV CheckMate 511 Trial. J. Clin. Oncol..

[25] Tawbi H.A., Schadendorf D., Lipson E.J., Ascierto P.A., Matamala L., Castillo Gutiérrez E., Rutkowski P., Gogas H.J., Lao C.D., De Menezes J.J. (2022). Relatlimab and Nivolumab versus Nivolumab in Untreated Advanced Melanoma. N. Engl. J. Med..

[26] Grob J.-J., Gonzalez R., Basset-Seguin N., Vornicova O., Schachter J., Joshi A., Meyer N., Grange F., Piulats J.M., Bauman J.R. (2020). Pembrolizumab Monotherapy for Recurrent or Metastatic Cutaneous Squamous Cell Carcinoma: A Single-Arm Phase II Trial (KEYNOTE-629). J. Clin. Oncol..

[27] Migden M.R., Khushalani N.I., Chang A.L.S., Lewis K.D., Schmults C.D., Hernandez-Aya L., Meier F., Schadendorf D., Guminski A., Hauschild A. (2020). Cemiplimab in locally advanced cutaneous squamous cell carcinoma: Results from an open-label, phase 2, single-arm trial. Lancet Oncol..

[28] Stratigos A.J., Sekulic A., Peris K., Bechter O., Prey S., Kaatz M., Lewis K.D., Basset-Seguin N., Chang A.L.S., Dalle S. (2021). Cemiplimab in locally advanced basal cell carcinoma after hedgehog inhibitor therapy: An open-label, multi-centre, single-arm, phase 2 trial. Lancet Oncol..

[29] Nghiem P.T., Bhatia S., Lipson E.J., Kudchadkar R.R., Miller N.J., Annamalai L., Berry S., Chartash E.K., Daud A., Fling S.P. (2016). PD-1 Blockade with Pembrolizumab in Advanced Merkel-Cell Carcinoma. N. Engl. J. Med..

[30] D’Angelo S.P., Russell J., Lebbé C., Chmielowski B., Gambichler T., Grob J.-J., Kiecker F., Rabinowits G., Terheyden P., Zwiener I. (2018). Efficacy and Safety of First-line Avelumab Treatment in Patients With Stage IV Metastatic Merkel Cell Carcinoma: A Preplanned Interim Analysis of a Clinical Trial. JAMA Oncol..

[31] Marthey L., Mateus C., Mussini C., Nachury M., Nancey S., Grange F., Zallot C., Peyrin-Biroulet L., Rahier J.F., Bourdier de Beauregard M. (2016). Cancer Immunotherapy with Anti-CTLA-4 Monoclonal Antibodies Induces an Inflammatory Bowel Disease. J. Crohn’s Colitis.

[32] Grover S., Dougan M., Tyan K., Giobbie-Hurder A., Blum S.M., Ishizuka J., Qazi T., Elias R., Vora K.B., Ruan A.B. (2020). Vitamin D intake is associated with decreased risk of immune checkpoint inhibitor-induced colitis. Cancer.

[33] Braga Neto M.B., Ramos G.P., Loftus E.V., Faubion W.A., Raffals L.E. (2021). Use of Immune Checkpoint Inhibitors in Patients With Pre-established Inflammatory Bowel Diseases: Retrospective Case Series. Clin. Gastroenterol. Hepatol..

[34] Grover S., Ruan A.B., Srivoleti P., Giobbie-Hurder A., Braschi-Amirfarzan M., Srivastava A., Buchbinder E.I., Ott P.A., Kehl K.L., Awad M.M. (2020). Safety of Immune Checkpoint Inhibitors in Patients With Pre-Existing Inflammatory Bowel Disease and Microscopic Colitis. JCO Oncol. Pract..

[35] Som A., Mandaliya R., Alsaadi D., Farshidpour M., Charabaty A., Malhotra N., Mattar M.C. (2019). Immune checkpoint inhibitor-induced colitis: A comprehensive review. World J. Clin. Cases.

[36] https://ctep.cancer.gov/protocoldevelopment/electronic_applications/docs/ctcae.

[37] Brahmer J.R., Abu-Sbeih H., Ascierto P.A., Brufsky J., Cappelli L.C., Cortazar F.B., Gerber D.E., Hamad L., Hansen E., Johnson D.B. (2021). Society for Immunotherapy of Cancer (SITC) clinical practice guideline on immune checkpoint inhibitor-related adverse events. J. ImmunoTherapy Cancer.

[38] Su H.-J., Chiu Y.-T., Chiu C.-T., Lin Y.-C., Wang C.-Y., Hsieh J.-Y., Wei S.-C. (2019). Inflammatory bowel disease and its treatment in 2018: Global and Taiwanese status updates. J. Formos. Med. Assoc..

[39] Ng S.C., Shi H.Y., Hamidi N., Underwood F.E., Tang W., Benchimol E.I., Panaccione R., Ghosh S., Wu J.C.Y., Chan F.K.L. (2017). Worldwide incidence and prevalence of inflammatory bowel disease in the 21st century: A systematic review of population-based studies. Lancet.

[40] McDowell C., Farooq U., MuhammadHaseeb M. (2022). Inflammatory Bowel Disease. [Updated 2022 May 1]. StatPearls [Internet].

[41] Shanahan F. (2001). Inflammatory bowel disease: Immunodiagnostics, immunotherapeutics, and ecotherapeutics. Gastroenterology.

[42] Podolsky D.K. (1991). Inflammatory Bowel Disease. N. Engl. J. Med..

[43] Baumgart D.C., Carding S.R. (2007). Inflammatory bowel disease: Cause and immunobiology. Lancet.

[44] Duchmann R., Kaiser I., Hermann E., Mayet W., Ewe K., Meyer zum Büschenfelde K.H. (1995). Tolerance exists towards resident intestinal flora but is broken in active inflammatory bowel disease (IBD). Clin. Exp. Immunol..

[45] de Zoeten E.F., Pasternak B.A., Mattei P., Kramer R.E., Kader H.A. (2013). Diagnosis and Treatment of Perianal Crohn Disease: NASPGHAN Clinical Report and Consensus Statement. J. Pediatr. Gastroenterol. Nutr..

[46] Maaser C., Sturm A., Vavricka S.R., Kucharzik T., Fiorino G., Annese V., Calabrese E., Baumgart D.C., Bettenworth D., Borralho Nunes P. (2018). ECCO-ESGAR Guideline for Diagnostic Assessment in IBD Part 1: Initial diagnosis, monitoring of known IBD, detection of complications. J. Crohn’s Colitis.

[47] Langner C., Magro F., Driessen A., Ensari A., Mantzaris G.J., Villanacci V., Becheanu G., Borralho Nunes P., Cathomas G., Fries W. (2014). The histopathological approach to inflammatory bowel disease: A practice guide. Virchows Arch..

[48] Best W.R., Becktel J.M., Singleton J.W., Kern F. (1976). Development of a Crohn’s disease activity index. National Cooperative Crohn’s Disease Study. Gastroenterology.

[49] Mary J.Y., Modigliani R. (1989). Development and validation of an endoscopic index of the severity for Crohn’s disease: A prospective multicentre study. Groupe d’Etudes Thérapeutiques des Affections Inflammatoires du Tube Digestif (GETAID). Gut.

[50] Schroeder K.W., Tremaine W.J., Ilstrup D.M. (1987). Coated oral 5-aminosalicylic acid therapy for mildly to moderately active ulcerative colitis. A randomized study. N. Engl. J. Med..

[51] Carter M.J., Lobo A.J., Travis S.P.L. (2004). Guidelines for the management of inflammatory bowel disease in adults. Gut.

[52] Prantera C., Viscido A., Biancone L., Francavilla A., Giglio L., Campieri M. (2005). A new oral delivery system for 5-ASA: Preliminary clinical findings for MMx. Inflamm. Bowel Dis..

[53] Robertson J., Haas C.T., Pele L.C., Monie T.P., Charalambos C., Parkes M., Hewitt R.E., Powell J.J. (2016). Intestinal APCs of the endogenous nanomineral pathway fail to express PD-L1 in Crohn’s disease. Sci. Rep..

[54] Lamb C.A., Kennedy N.A., Raine T., Hendy P.A., Smith P.J., Limdi J.K., Hayee B.H., Lomer M.C.E., Parkes G.C., Selinger C. (2019). British Society of Gastroenterology consensus guidelines on the management of inflammatory bowel disease in adults. Gut.

[55] Hanauer S.B., Strömberg U. (2004). Oral Pentasa in the treatment of active Crohn’s disease: A meta-analysis of double-blind, placebo-controlled trials. Clin. Gastroenterol. Hepatol..

[56] Prefontaine E., Sutherland L.R., Macdonald J.K., Cepoiu M. (2009). Azathioprine or 6-mercaptopurine for maintenance of remission in Crohn’s disease. Cochrane Database Syst. Rev..

[57] Timmer A., McDonald J.W., Tsoulis D.J., Macdonald J.K. (2012). Azathioprine and 6-mercaptopurine for maintenance of remission in ulcerative colitis. Cochrane Database Syst. Rev..

[58] Lv R., Qiao W., Wu Z., Wang Y., Dai S., Liu Q., Zheng X. (2014). Tumor necrosis factor alpha blocking agents as treatment for ulcerative colitis intolerant or refractory to conventional medical therapy: A meta-analysis. PLoS ONE.

[59] van Deventer S.J.H. (1999). Anti-TNF antibody treatment of Crohn’s disease. Ann. Rheum. Dis..

[60] Sandborn W.J., Su C., Sands B.E., D’Haens G.R., Vermeire S., Schreiber S., Danese S., Feagan B.G., Reinisch W., Niezychowski W. (2017). Tofacitinib as Induction and Maintenance Therapy for Ulcerative Colitis. N. Engl. J. Med..

[61] Danese S., Roda G., Peyrin-Biroulet L. (2020). Evolving therapeutic goals in ulcerative colitis: Towards disease clearance. Nat. Rev. Gastroenterol. Hepatol..

[62] Lichtenstein G.R., Rutgeerts P. (2009). Importance of mucosal healing in ulcerative colitis. Inflamm. Bowel Dis..

[63] Bryant R.V., Winer S., SPL T., Riddell R.H. (2014). Systematic review: Histological remission in inflammatory bowel disease. Is ‘complete’ remission the new treatment paradigm? An IOIBD initiative. J. Crohn’s Colitis.

[64] Yoon H., Jangi S., Dulai P.S., Boland B.S., Jairath V., Feagan B.G., Sandborn W.J., Singh S. (2021). Histologic Remission Is Associated With Lower Risk of Treatment Failure in Patients With Crohn Disease in Endoscopic Remission. Inflamm. Bowel Dis..

[65] Peyrin-Biroulet L., Bressenot A., Kampman W. (2014). Histologic remission: The ultimate therapeutic goal in ulcerative colitis?. Clin. Gastroenterol. Hepatol..

[66] Riley S.A., Mani V., Goodman M.J., Dutt S., Herd M.E. (1991). Microscopic activity in ulcerative colitis: What does it mean?. Gut.

[67] Thompson J.A., Schneider B.J., Brahmer J., Achufusi A., Armand P., Berkenstock M.K., Bhatia S., Budde L.E., Chokshi S., Davies M. (2022). Management of Immunotherapy-Related Toxicities, Version 1.2022, NCCN Clinical Practice Guidelines in Oncology. J. Natl. Compr. Cancer Netw..

[68] Sharma P., Siddiqui B.A., Anandhan S., Yadav S.S., Subudhi S.K., Gao J., Goswami S., Allison J.P. (2021). The Next Decade of Immune Checkpoint Therapy. Cancer Discov..

[69] Chang J.T. (2020). Pathophysiology of Inflammatory Bowel Diseases. N. Engl. J. Med..

[70] Zhang M., Ni J., Xu W.D., Wen P.F., Qiu L.J., Wang X.S., Pan H.F., Ye D.Q. (2014). Association of CTLA-4 variants with susceptibility to inflammatory bowel disease: A meta-analysis. Hum. Immunol..

[71] Ni J., Qiu L.-J., Zhang M., Wen P.-F., Ye X.-R., Liang Y., Pan H.-F., Ye D.-Q. (2014). CTLA-4 CT60 (rs3087243) polymorphism and autoimmune thyroid diseases susceptibility: A comprehensive meta-analysis. Endocr. Res..

[72] Schwab C., Gabrysch A., Olbrich P., Patiño V., Warnatz K., Wolff D., Hoshino A., Kobayashi M., Imai K., Takagi M. (2018). Phenotype, penetrance, and treatment of 133 cytotoxic T-lymphocyte antigen 4-insufficient subjects. J. Allergy Clin. Immunol..

[73] Zamani M.R., Aslani S., Salmaninejad A., Javan M.R., Rezaei N. (2016). PD-1/PD-L and autoimmunity: A growing relationship. Cell Immunol..

[74] Powell J.J., Thomas-McKay E., Thoree V., Robertson J., Hewitt R.E., Skepper J.N., Brown A., Hernandez-Garrido J.C., Midgley P.A., Gomez-Morilla I. (2015). An endogenous nanomineral chaperones luminal antigen and peptidoglycan to intestinal immune cells. Nat. Nanotechnol..

[75] Choi K., Abu-Sbeih H., Samdani R., Nogueras Gonzalez G., Raju G.S., Richards D.M., Gao J., Subudhi S., Stroehlein J., Wang Y. (2019). Can Immune Checkpoint Inhibitors Induce Microscopic Colitis or a Brand New Entity?. Inflamm. Bowel Dis..

[76] Hone Lopez S., Kats-Ugurlu G., Renken R.J., Buikema H.J., de Groot M.R., Visschedijk M.C., Dijkstra G., Jalving M., de Haan J.J. (2021). Immune checkpoint inhibitor treatment induces colitis with heavy infiltration of CD8 + T cells and an infiltration pattern that resembles ulcerative colitis. Virchows Arch..

[77] Geukes Foppen M.H., Rozeman E.A., van Wilpe S., Postma C., Snaebjornsson P., van Thienen J.V., van Leerdam M.E., van den Heuvel M., Blank C.U., van Dieren J. (2018). Immune checkpoint inhibition-related colitis: Symptoms, endoscopic features, histology and response to management. ESMO Open.

[78] National Comprehensive Cancer, N Version 1.2022—28 February 2022. https://www.nccn.org/professionals/physician_gls/pdf/immunotherapy.pdf.

[79] Zou F., Wang X., Glitza Oliva I.C., McQuade J.L., Wang J., Zhang H.C., Thompson J.A., Thomas A.S., Wang Y. (2021). Fecal calprotectin concentration to assess endoscopic and histologic remission in patients with cancer with immune-mediated diarrhea and colitis. J. ImmunoTherapy Cancer.

[80] Reddavide R., Rotolo O., Caruso M.G., Stasi E., Notarnicola M., Miraglia C., Nouvenne A., Meschi T., De’ Angelis G.L., Di Mario F. (2018). The role of diet in the prevention and treatment of Inflammatory Bowel Diseases. Acta Biomed..

[81] Voitk A.J., Echave V., Feller J.H., Brown R.A., Gurd F.N. (1973). Experience with elemental diet in the treatment of inflammatory bowel disease. Is this primary therapy?. Arch. Surg..

[82] Shafiee N.H., Manaf Z.A., Mokhtar N.M., Raja Ali R.A. (2021). Anti-inflammatory diet and inflammatory bowel disease: What clinicians and patients should know?. Intest. Res..

[83] Ricour C., Duhamel J.F., Nihoul-Fekete C. (1977). Use of parenteral and elementary enteral nutrition in the treatment of Crohn’s disease and ulcerative colitis in children. Arch. Fr. De Pediatr..

[84] Lochs H., Steinhardt H.J., Klaus-Wentz B., Zeitz M., Vogelsang H., Sommer H., Fleig W.E., Bauer P., Schirrmeister J., Malchow H. (1991). Comparison of enteral nutrition and drug treatment in active Crohn’s disease. Results of the European Cooperative Crohn’s Disease Study. IV. Gastroenterology.

[85] Green N., Miller T., Suskind D., Lee D. (2019). A Review of Dietary Therapy for IBD and a Vision for the Future. Nutrients.

[86] Nazarian L.F. (1997). A Synopsis of the American Academy of Pediatrics’Practice Parameter on the Management of Acute Gastroenteritis in Young Children. Pediatr. Rev..

[87] Hwang C., Ross V., Mahadevan U. (2014). Popular exclusionary diets for inflammatory bowel disease: The search for a dietary culprit. Inflamm. Bowel Dis..

[88] Lau T.C., Fiebig-Comyn A.A., Shaler C.R., McPhee J.B., Coombes B.K., Schertzer J.D. (2021). Low dietary fiber promotes enteric expansion of a Crohn’s disease-associated pathobiont independent of obesity. Am. J. Physiol. Endocrinol. Metab..

[89] Konijeti G.G., Kim N., Lewis J.D., Groven S., Chandrasekaran A., Grandhe S., Diamant C., Singh E., Oliveira G., Wang X. (2017). Efficacy of the Autoimmune Protocol Diet for Inflammatory Bowel Disease. Inflamm. Bowel Dis..

[90] Gibson P.R., Shepherd S.J. (2005). Personal view: Food for thought--western lifestyle and susceptibility to Crohn’s disease. The FODMAP hypothesis. Aliment. Pharmacol. Ther..

[91] Prince A.C., Myers C.E., Joyce T., Irving P., Lomer M., Whelan K. (2016). Fermentable Carbohydrate Restriction (Low FODMAP Diet) in Clinical Practice Improves Functional Gastrointestinal Symptoms in Patients with Inflammatory Bowel Disease. Inflamm. Bowel Dis..

[92] Chiba M., Abe T., Tsuda H., Sugawara T., Tsuda S., Tozawa H., Fujiwara K., Imai H. (2010). Lifestyle-related disease in Crohn’s disease: Relapse prevention by a semi-vegetarian diet. World J. Gastroenterol..

[93] Chicco F., Magri S., Cingolani A., Paduano D., Pesenti M., Zara F., Tumbarello F., Urru E., Melis A., Casula L. (2021). Multidimensional Impact of Mediterranean Diet on IBD Patients. Inflamm. Bowel Dis..

[94] Gottschall E.G. (1994). Breaking the Vicious Cycle: Intestinal Health through Diet.

[95] Sandefur K., Kahleova H., Desmond A.N., Elfrink E., Barnard N.D. (2019). Crohn’s Disease Remission with a Plant-Based Diet: A Case Report. Nutrients.

[96] Obih C., Wahbeh G., Lee D., Braly K., Giefer M., Shaffer M.L., Nielson H., Suskind D.L. (2016). Specific carbohydrate diet for pediatric inflammatory bowel disease in clinical practice within an academic IBD center. Nutrition.

[97] Lewis J.D., Sandler R.S., Brotherton C., Brensinger C., Li H., Kappelman M.D., Daniel S.G., Bittinger K., Albenberg L., Valentine J.F. (2021). A Randomized Trial Comparing the Specific Carbohydrate Diet to a Mediterranean Diet in Adults With Crohn’s Disease. Gastroenterology.

[98] Hou J.K., Lee D., Lewis J. (2014). Diet and inflammatory bowel disease: Review of patient-targeted recommendations. Clin. Gastroenterol. Hepatol..

[99] Nishiguchi R., Basu S., Staab H.A., Ito N., Zhou X.K., Wang H., Ha T., Johncilla M., Yantiss R.K., Montrose D.C. (2021). Dietary interventions to prevent high-fructose diet-associated worsening of colitis and colitis-associated tumorigenesis in mice. Carcinogenesis.

[100] Tan R., Dong H., Chen Z., Jin M., Yin J., Li H., Shi D., Shao Y., Wang H., Chen T. (2021). Intestinal Microbiota Mediates High-Fructose and High-Fat Diets to Induce Chronic Intestinal Inflammation. Front. Cell. Infect. Microbiol..

[101] Chiba M., Nakane K., Tsuji T., Tsuda S., Ishii H., Ohno H., Watanabe K., Ito M., Komatsu M., Yamada K. (2018). Relapse Prevention in Ulcerative Colitis by Plant-Based Diet Through Educational Hospitalization: A Single-Group Trial. Perm. J..

[102] Niewiadomski O., Studd C., Wilson J., Williams J., Hair C., Knight R., Prewett E., Dabkowski P., Alexander S., Allen B. (2016). Influence of food and lifestyle on the risk of developing inflammatory bowel disease. Intern. Med. J..

[103] Weisshof R., Chermesh I. (2015). Micronutrient deficiencies in inflammatory bowel disease. Curr. Opin. Clin. Nutr. Metab. Care.

[104] Fletcher J., Cooper S.C., Ghosh S., Hewison M. (2019). The Role of Vitamin D in Inflammatory Bowel Disease: Mechanism to Management. Nutrients.

[105] Raman M., Milestone A.N., Walters J.R., Hart A.L., Ghosh S. (2011). Vitamin D and gastrointestinal diseases: Inflammatory bowel disease and colorectal cancer. Ther. Adv. Gastroenterol..

[106] Dresner-Pollak R., Ackerman Z., Eliakim R., Karban A., Chowers Y., Fidder H.H. (2004). The BsmI Vitamin D Receptor Gene Polymorphism Is Associated with Ulcerative Colitis in Jewish Ashkenazi Patients. Genet. Test..

[107] Cantorna M.T., Munsick C., Bemiss C., Mahon B.D. (2000). 1,25-Dihydroxycholecalciferol prevents and ameliorates symptoms of experimental murine inflammatory bowel disease. J. Nutr..

[108] Li J., Chen N., Wang D., Zhang J., Gong X. (2018). Efficacy of vitamin D in treatment of inflammatory bowel disease: A meta-analysis. Medicine.

[109] Uchiyama K., Nakamura M., Odahara S., Koido S., Katahira K., Shiraishi H., Ohkusa T., Fujise K., Tajiri H. (2010). N-3 polyunsaturated fatty acid diet therapy for patients with inflammatory bowel disease. Inflamm. Bowel Dis..

[110] Cabré E., Mañosa M., Gassull M.A. (2012). Omega-3 fatty acids and inflammatory bowel diseases—A systematic review. Br. J. Nutr..

[111] Feagan B.G., Sandborn W.J., Mittmann U., Bar-Meir S., D’Haens G., Bradette M., Cohen A., Dallaire C., Ponich T.P., McDonald J.W.D. (2008). Omega-3 Free Fatty Acids for the Maintenance of Remission in Crohn Disease: The EPIC Randomized Controlled Trials. JAMA.

[112] Damas O.M., Garces L., Abreu M.T. (2019). Diet as Adjunctive Treatment for Inflammatory Bowel Disease: Review and Update of the Latest Literature. Curr. Treat. Opt. Gastroenterol..

[113] Simadibrata M., Halimkesuma C.C., Suwita B.M. (2017). Efficacy of Curcumin as Adjuvant Therapy to Induce or Maintain Remission in Ulcerative Colitis Patients: An Evidence-based Clinical Review. Acta Med. Indones..

[114] Banerjee R., Penmetsa A., Medaboina K., Boramma G.G., Amsrala S., Reddy D.N. (2017). Novel Bio-Enhanced Curcumin with Mesalamine for Induction of Remission in Mild to Moderate Ulcerative Colitis. Gastroenterology.

[115] Wu G.D., Chen J., Hoffmann C., Bittinger K., Chen Y.Y., Keilbaugh S.A., Bewtra M., Knights D., Walters W.A., Knight R. (2011). Linking long-term dietary patterns with gut microbial enterotypes. Science.

[116] Ma X., Torbenson M., Hamad A.R., Soloski M.J., Li Z. (2008). High-fat diet modulates non-CD1d-restricted natural killer T cells and regulatory T cells in mouse colon and exacerbates experimental colitis. Clin. Exp. Immunol..

[117] Pitcher M.C., Cummings J.H. (1996). Hydrogen sulphide: A bacterial toxin in ulcerative colitis?. Gut.

[118] Spencer C.N., McQuade J.L., Gopalakrishnan V., McCulloch J.A., Vetizou M., Cogdill A.P., Khan M.A.W., Zhang X., White M.G., Peterson C.B. (2021). Dietary fiber and probiotics influence the gut microbiome and melanoma immunotherapy response. Science.

[119] Mentella M.C., Scaldaferri F., Pizzoferrato M., Gasbarrini A., Miggiano G.A.D. (2020). Nutrition, IBD and Gut Microbiota: A Review. Nutrients.

[120] Dubrovsky A., Kitts C.L. (2018). Effect of the Specific Carbohydrate Diet on the Microbiome of a Primary Sclerosing Cholangitis and Ulcerative Colitis Patient. Cureus.

[121] Vervier K., Moss S., Kumar N., Adoum A., Barne M., Browne H., Kaser A., Kiely C.J., Neville B.A., Powell N. (2022). Two microbiota subtypes identified in irritable bowel syndrome with distinct responses to the low FODMAP diet. Gut.

[122] Tomova A., Bukovsky I., Rembert E., Yonas W., Alwarith J., Barnard N.D., Kahleova H. (2019). The Effects of Vegetarian and Vegan Diets on Gut Microbiota. Front. Nutr..

[123] Vétizou M., Pitt J.M., Daillère R., Lepage P., Waldschmitt N., Flament C., Rusakiewicz S., Routy B., Roberti M.P., Duong C.P. (2015). Anticancer immunotherapy by CTLA-4 blockade relies on the gut microbiota. Science.

[124] Sivan A., Corrales L., Hubert N., Williams J.B., Aquino-Michaels K., Earley Z.M., Benyamin F.W., Lei Y.M., Jabri B., Alegre M.L. (2015). Commensal Bifidobacterium promotes antitumor immunity and facilitates anti-PD-L1 efficacy. Science.

[125] Gopalakrishnan V., Spencer C.N., Nezi L., Reuben A., Andrews M.C., Karpinets T.V., Prieto P.A., Vicente D., Hoffman K., Wei S.C. (2018). Gut microbiome modulates response to anti-PD-1 immunotherapy in melanoma patients. Science.

